# Balancing act: influence of Cu content in NiCu/C catalysts for methane decomposition†

**DOI:** 10.1039/d4ma00138a

**Published:** 2024-03-30

**Authors:** Suzan E. Schoemaker, Stefan Bismeijer, Dennie F. L. Wezendonk, Johannes D. Meeldijk, Tom A. J. Welling, Petra E. de Jongh

**Affiliations:** a Materials Chemistry and Catalysis, Debye Institute for Nanomaterial Science, Universiteit Utrecht Universiteitsweg 99 3584 CG Utrecht The Netherlands t.a.j.welling@uu.nl p.e.dejongh@uu.nl; b Electron Microscopy Center, Faculty of Science, Universiteit Utrecht Universiteitsweg 99 3584 CG Utrecht The Netherlands

## Abstract

Thermal catalytic decomposition of methane is an innovative pathway to produce CO_2_-free hydrogen from natural gas. We investigated the role of Cu content in carbon-supported bimetallic NiCu catalysts. A graphitic carbon material was used as a model support, and we combined operando methane decomposition experiments in a thermogravimetric analyzer with *in situ* electron microscopy measurements. The carbon yield was maximum with around 30% Cu in the nanoparticles. Adding more Cu drastically lowered the carbon solubility in the metal nanoparticles, which lowered the initial reaction rate and overall carbon yield. *In situ* TEM measurements showed that the addition of Cu to the catalysts strongly influenced the metal nanoparticle shape and size during carbon growth, and the growth mode. NiCu particles were larger, remained spherical and facilitated steady CNF growth. In contrast, pure Ni nanoparticles fluctuated in shape, sometimes fragmented, and showed stuttering CNF growth. This was ascribed to fluctuating coverage of part of the Ni nanoparticle surface with amorphous carbon, which increased the chance of total encapsulation and hence deactivation of the individual Ni nanoparticles. This supports a picture where balancing the carbon supply, transport, and nucleation of amorphous and crystalline carbon is crucial. Our results also highlight the importance of combining statistically relevant measurements with microscopic information on individual nanoparticles to understand overall catalytic trends from the combined behavior of individual catalyst nanoparticles.

## Abstract 2

Thermal catalytic decomposition of methane is an innovative pathway to produce CO_2_-free hydrogen from natural gas. Initial growth rate correlated with carbon solubility in the metal catalyst.

## Graphical abstract

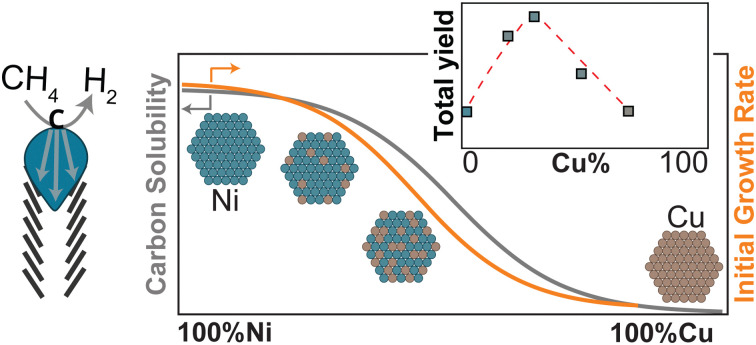

## 1. Introduction

A route to sustainable hydrogen production is *via* the thermocatalytic decomposition of methane, which produces pure hydrogen gas as well as solid carbon nanomaterials (carbon nanofibers or tubes).^1–4^ These carbon nanostructures can be used for example for battery materials, electrode materials, construction materials, or catalyst supports.^5–8^

The reaction can be divided into three main steps: (1) methane dissociation – carbon supply, (2) carbon diffusion typically through the metal nanoparticle – carbon transport, and (3) carbon nanostructure formation.^9,10^ However, next to the desired crystalline carbon also undesired carbon deposition can take place, which can deactivate the metal nanoparticle by encapsulation. The balance between these steps determines how long the reaction can proceed without catalyst deactivation as well as the final carbon nanostructures that are formed.^11^ Many parameters influence the reaction, *e.g.* reaction conditions (temperature and feedstock) and the catalyst composition and structure (metal, particle size, *etc.*).^1,12–15^ Depending on the operating temperature, the rate-determining step can be methane dissociation or the nucleation of and growth of ordered carbon structures, while the main reaction product can shift with increasing temperature from full, solid fibers to hollow fibers.^10^

Typically, supported Ni nanoparticles are used as growth catalysts, due to their high activity and high carbon solubility.^16–22^ However, a disadvantage is that these catalysts deactivate quickly. To overcome this problem, the use of bimetallic catalysts has been widely explored. Especially, bimetallic Ni–Cu catalysts have received much attention.^10,14,15,23–33^ NiCu catalysts usually provide higher activities and longer lifetimes than Ni catalysts and their use increases the optimal reaction temperature – which allows for higher conversions.^29,31^ However, the exact role of Cu and why it is so beneficial is not clear yet, especially not its influence on the individual nanoparticle level.

Copper catalysts are utilized for graphene growth with chemical vapor deposition, which is typically performed at temperatures between 800 and 1000 °C.^34,35^ Yet, due to the filled d-band of Cu, it is much less active for methane decomposition than Ni. Therefore, the role of Cu must be due to altering the properties of the catalytic metal nanoparticle. Suelves *et al.* and others reported that a dilution effect by the presence of Cu could improve methane adsorption and hence carbon fiber growth.^15,17,23,26^ Promoting carbon diffusion by an increased lattice constant was also postulated.^23^ Carbon solubility might be important and is greatly impacted by changes in the electronic structure.^9,36,37^ Xu *et al.* reported stabilization of larger particles as a cause for the improved activity^27^ since they facilitate a more steady growth.^27,38^ Turaeva *et al.* noticed that the electronic structure, especially the position of the top of the d-band with respect to the Fermi level is of key importance for nanostructure growth,^9^ which is changed by alloying Ni with Cu.^39^

To understand overall macroscopic observations on the influence of catalyst composition on catalytic performance, it is essential to also have a closer look at the individual particles.^38^ In this work, we will dive deeper into the role of Cu, combining a thermogravimetric analyzer to follow carbon growth under operando conditions with *in situ* electron microscopy to understand the impact of composition on the behavior of individual nanoparticles. We studied a series of Ni-based catalysts with varying Cu content (0–62%), using graphitic carbon as a model support to limit metal–support interaction.^40,41^ This allowed us to demonstrate that the CNF growth modes of Ni and NiCu particles differ greatly, and how the rates of different steps have to be balanced to obtain a maximum carbon yield.

## 2. Experimental

### 2.1. Catalyst preparation

For this study, catalysts were prepared using incipient wetness (co-)impregnation. First, 4 M stock solutions were made by dissolving Ni(NO_3_)_2_·6H_2_O (≥97%, Sigma Aldrich) or Cu(NO_3_)_2_·3H_2_O (99%, Sigma Aldrich) in Mili Q water. The solutions were acidified with HNO_3_ to obtain a pH ∼ 1, well below the point-of-zero charge of the carbon support (∼4) hence optimizing electrostatic interaction between the positively charged support surface and the negatively charged nickel nitrate complex. These stock solutions were combined to obtain the desired ratios for preparing the different catalysts. A carbon support (xGnPC-500, XG Sciences) was impregnated (90% of the pore volume, *V*_p,total_ = 0.87 mL g^−1^) with the precursor solutions. The carbon support consisted of thin carbon sheets, graphene nanoplatelets, with a surface area of ∼500 m^2^ g^−1^. The support was used as received without any modification. Table 1 gives an overview of the preparation details of a series of five catalysts. After impregnation, the samples were dried under dynamic vacuum at room temperature overnight. For the sequential impregnation, the sample was dried under dynamic vacuum at room temperature in between the two impregnation cycles and dried again after the second cycle. The metal nitrates were decomposed into oxides during heat treatment at 330 °C in N_2_ (200 mL min^−1^ g^−1^) for 2 hours. Finally, the catalysts were reduced at 350 (catalyst 1), 330 (catalyst 2) or 280 (catalysts 3–5) °C in 5% H_2_/Ar for 3 hours (5 °C min^−1^).

**Table 1:** Preparation conditions catalysts

Catalyst		Precursor (M)		Method	Heat treatment (°C)	Reduction (°C)
		Ni	Cu			
1	Ni/C	3	—	IWI	330	350
2	Ni_84_Cu_16_/C	3	0.5	co-IWI		330
3	Ni_74_Cu_26_/C	3	1	co-IWI		280
4	Ni_56_Cu_44_/C	2.4	1.6	co-IWI		280
5	Ni_38_Cu_62_/C	1	1.5	Seq-co-IWI		280

### 2.2. Methane decomposition in a thermogravimetric analyzer

The methane decomposition experiments were performed in a thermogravimetric analyzer (TGA, TGA5500, TA Instruments) with an IR furnace connected to a mass spectrometer (Discovery II MS, TA instruments). The gas feed flows horizontally over the catalyst bed, the catalytic setup is shown in Fig. S1 (ESI†).

Before starting the experiments, the system was purged with Ar (100 mL min^−1^) for 1 or 2 hours, to remove the oxygen from the sample chamber. Thereafter, the sample was dried at 70 °C in Ar (50 mL min^−1^) for 15 min. This was followed by *in situ* reduction at 350 (Ni/C), 330 (2) or 300 (3–5) °C in 5% H_2_/Ar for 75 min (5 °C min^−1^).

Then, the sample was heated up to reaction temperature (5 °C min^−1^). After 2 min equilibration at reaction temperature, the reaction gas was introduced. The experiments were performed with 30% CH_4_ in Ar (total flow 50 mL min^−1^) at 500 and 600 °C. No other gaseous products other than H_2_ or (residual) CH_4_ were detected during the experiments.

All experiments were performed in duplo. To correct for the difference in metal loading all measurements were normalized per gram of metal (Ni plus Cu). The differences between the duplo measurements were below 10%. Examples of the duplo measurements are shown in Fig. S2 (ESI†).

The growth rate was calculated by taking the derivative of the carbon accumulation with respect to time. The initial growth rate therefore is taken as the first point of the derivative.

### 2.3. Methane decomposition using *in situ* transmission electron microscopy

The gas-cell transmission electron microscopy (TEM) experiments were performed on a Talos F200X (Thermo-Fischer Scientific), equipped with a field-emission gun operated in TEM mode at 200 kV, roughly following the methodology described in our previous work.^38^ The electron dose rate was kept below 10 e^−^ Å^−2^ s^−1^, to limit the influence of the electron beam.^38^ A 40-μm objective aperture was used. A 4k × 4k Ceta camera was used to acquire bright-field TEM images, with an exposure time of 1 or 2 s. Image series were recorded with a pixel size of approximately 1 nm with 2048 by 2048 pixels.

The *in situ* TEM experiments were performed in a dedicated gas-cell system (Protochips Atmosphere 210) including a sample holder, gas supply system, and a heating control unit. The cell within the sample holder consists of a top and bottom chip, which were joined using o-rings to separate the airtight inner cell compartment from the high vacuum of the microscope. One of the chips contained a silicon carbide-based heating membrane used for closed-loop temperature control using the resistance of the silicon carbide. Both chips contained six 30–50 nm thick silicon nitride windows which allowed imaging with the electron beam while containing the gas within the cell. The gas supply system had tanks in which gases could be mixed before they were flowed towards the sample holder.

The two chips were glow-discharged (Cressington Power Unit 208) for 30 seconds prior to the experiment. Then a dilute pre-reduced catalyst in ethanol was dropcast approximately 20 times (0.5 μL per time) on the chip containing the heating membrane. Subsequently, the cell was assembled in the dedicated holder and checked for potential leaks. After the holder was inserted into the microscope, it was first flushed with Ar for 5 minutes at 0.1 sccm at 1 bar. Subsequently, an *in situ* reduction step in 5% H_2_ and 95% Ar at 1 bar (0.1 sccm) at 300 °C was performed for 45 minutes. The temperature was then increased to the reaction temperature (600 °C) at 2 °C s^−1^. The reaction gas (10% H_2_, 30% CH_4_, and 60% Ar at 1 bar, 0.1 sccm) was introduced at the moment that 600 °C was reached.

Data analysis was performed in ImageJ (version 1.53c). To measure the length of the carbon fibers, we identified a fiber of which the length could be reliably measured. This means that there was a reference point on the fiber that did not move during the measurement time. The length that the fibers had grown in a time frame of 15 to 30 s was measured. The change in length divided by the time between the first and next frame was used to calculate the average CNF growth rate for that particle. The particles often changed growth direction within this time frame, which led to CNFs that were not straight. In those cases, the distance from the reference feature to the first corner was measured, followed by the distances between corners, and lastly, the distance between the last corner and the particle, using the segmented-line feature in ImageJ. Sometimes multiple carbon structures grew from the same particle at the same time. The sum of the changes in length of all these structures was then taken to determine the carbon structure length growth rate from that specific metal particle.

### 2.4. Characterization of the fresh and used catalysts

H_2_-Temperature Programmed Reduction (TPR) was performed on an AutoChem II 2920 apparatus (Micromeritics). ∼50 mg heat-treated catalyst (sieved fraction 38–75 μm) was loaded in a U-shape tube in between quartz wool. First, the sample was dried at 120 °C. Thereafter, the TPR profile was measured from 30–500 °C (5 °C min^−1^) in 5% H_2_/Ar (flow = 40 mL min^−1^). A thermal conductivity detector (TCD) was used to obtain the reduction profiles. To check for support methanation, an MS signal was recorded on a Hiden Analytical mass spectrometer.

N_2_-physisorption at 77 K was performed to determine the BET surface areas and pore volumes of the carbon support and the reduced catalysts. This was done on a Tristar II Plus apparatus (Micromeritics). The samples were dried overnight under vacuum at 170 °C before analysis. The BET surface area was determined in the relative pressure range of *p*/*p*_0_ = 0.02–0.1. The total pore volume was determined from the adsorbed quantity at *p*/*p*_0_ = 0.995. Results are shown in Section S3 of the ESI.†

Powder X-ray Diffraction (XRD) was performed on a Bruker D2 Phaser 2nd Generation diffractometer with a Co radiation source (*λ* = 1.7889 Å). A sealed dome was used to measure the reduced catalysts under inert atmosphere. A Thermo-Fischer Scientific Talos F200X was operated at 200 kV in TEM mode to capture bright-field images of the catalyst. Scanning transmission electron microscopy Energy dispersive X-ray spectroscopy (STEM-EDX) mapping was used to map the distribution of Ni and Cu in the catalysts before catalysis. ICP analysis of the metal weight loadings was performed by the Mikroanalytisches Laboratorium Kolbe.

## 3. Results and discussion

### 3.1. Catalyst structural properties

A series of Ni-based catalysts containing 0–62% Cu was synthesized. The metal weight loadings were determined with ICP and are listed in Table 2. Note that since the Ni weight loading was kept constant, the total metal weight loading varies – for example the total metal loading in the Ni_38_Cu_62_ is 26% while the metal loading of Ni_84_Cu_16_ is 12.8%. The total surface area and pore volume of the reduced catalysts were determined based on N_2_-physisorption isotherms (Fig. S3, ESI†). The shape of the isotherms is similar for all catalysts indicating that the support structure was not significantly affected by the catalyst preparation. The BET surface area of the catalysts with 0–44% Cu are similar (250–270 m^2^ g^−1^), but the surface area of the catalyst containing 62% Cu is lower (174 m^2^ g^−1^). This is probably a result of the higher total metal loading of this catalyst. The total pore volume ranges between 0.30–0.48 mL g^−1^ (Table S3.1, ESI†).

**Table 2:** Catalyst properties

Name	Weight loading^a^ (%)			Av. Cu% per particle^a^	Particle size fresh catalyst^b^	
	Ni	Cu	Total metal		500 °C	600 °C
Ni/C	14.9	—	14.9	0	7.7 ± 2.5	—
Ni_84_Cu_16_/C	10.8	2	12.8	16	10.2 ± 2.0	12.6 ± 3.0
Ni_74_Cu_26_/C	11.7	4.1	15.8	26	11.0 ± 4.0	14.3 ± 5.0
Ni_56_Cu_44_/C	9.9	7	16.9	44	10.9 ± 3.0	14 ± 4.0
Ni_38_Cu_62_/C	9.9	16	26	62	10.4 ± 4.5	14.7 ± 6.0

a Based on ICP.

b Number-averaged particle size (diameter) as determined using TEM.

**Fig. 1:**
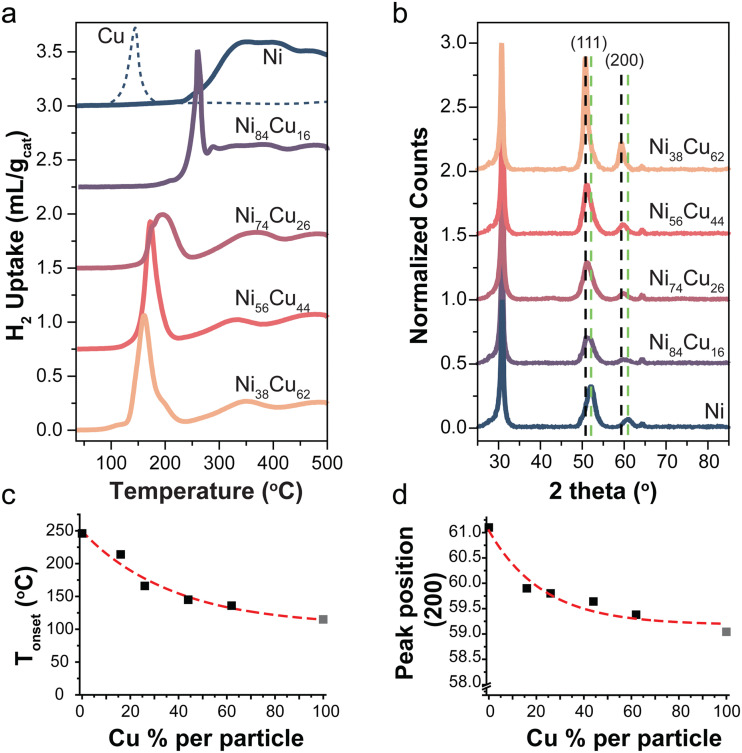
(a) Reduction profiles of selected catalysts with different Ni : Cu ratios (5 °C min^−1^ in 5% H_2_). A Cu reference shown in dotted line. (b) X-ray diffractograms of the reduced catalysts normalized to the carbon peak at 30°. Dotted lines indicate the reflections of the (111) and (200) planes of metallic Cu (black) and Ni (green). (c) The onset temperature of first reduction peak as function of Cu content. Data point of 100% Cu (gray) was taken from literature,^10^ the reduction profile is shown in Fig. S3.3b (ESI†). (d) Peak position of the (200) peak as a function of the Cu content. Peak position for 100% metallic Cu (gray) was obtained from ref. 46.

The reduction profiles of a series selected catalysts are shown in Fig. 1a. For the monometallic Ni-catalyst, multiple peaks are observed which can be ascribed to the reduction of NiO to Ni, weakly or more strongly interacting with the support.^42,43^ The reduction profile of NiO changes by introducing Cu in the system. A Cu/C reference is shown in the dashed line. Fig. 1c shows the onset temperature of the reduction as a function of Cu content. There is a peak shift to lower temperatures with increasing Cu content in the catalysts, consistent with what is known from literature.^23^ This is in line with Ni and Cu being present as an alloy.^10,44,45^ Starting from 300 °C support methanation plays a role for all catalysts,^10,40^ an example of the corresponding methane MS signal for the reduction profile of the Ni/C catalyst is shown in Fig. S4a (ESI†). By monitoring the sample weight during *in situ* reduction in the TGA (Fig. S5, ESI†), we confirmed that the reduction was completed before starting catalysis.

X-ray diffraction patterns of the selected reduced catalysts are shown in Fig. 1b. There is no indication of NiO or Cu_*x*_O in the samples, meaning that the samples are fully reduced. Diffraction peaks at 2*θ* = 30, 52, and 64° are attributed to the graphite support.^46^ The dotted lines indicate the (111) and (200) reflections for metallic Ni (green) and Cu (black). Fig. 1d shows the peak maximum of the (200) peak as a function of Cu content. This peak was used as it does not overlap with the carbon support peaks. The (200) peak in the bimetallic particles is found at intermediate positions with respect to pure Ni and Cu. This is again in line with alloy formation.^29,47^ Note that the intensity of the peaks varies for the different catalysts due to different total metal weight loadings (Table 2).

**Fig. 2:**
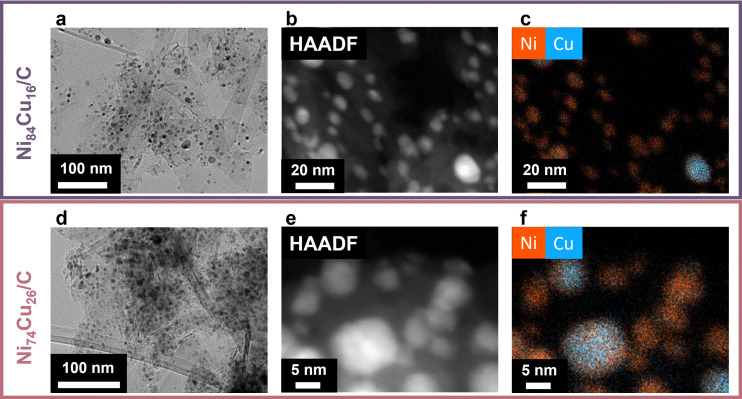
Transmission electron microscopy images with low magnification (left), HAADF STEM (middle) and corresponding EDX maps (right) of the fresh Ni_84_Cu_16_ (top, a–c) and Ni_74_Cu_26_ (bottom, d–f) catalysts after heating to reaction temperature of 500 °C in Ar.

Transmission electron microscopy (TEM) was utilized to assess the size and dispersion of the catalyst particles (Fig. 2). Fig. 2a and d displays the fresh Ni_84_Cu_16_ and Ni_74_Cu_26_ catalysts. Additional images of these catalysts, heated to 600 °C, along with images of the other fresh catalysts, are presented in Fig. S6–S8 (ESI†). The average particle sizes are summarized in Table 2. Details on the particle size distributions are available in the ESI† (Fig. S6 and S9). The average particle size of the Ni/C catalyst is 7.7 nm, somewhat smaller than that of the NiCu/C catalysts, which range from 10 to 11 nm. Previous research indicates that NiCu particles are mobile and susceptible to sintering during pre-treatment processes.^10^ Therefore, particle sizes were documented at both 500 °C and 600 °C prior to initiating the reaction.

Fig. 2b, c, e and f shows high magnification scanning transmission high angle annular dark field electron micrographs (Fig. 2b and e) along with corresponding energy dispersive X-ray spectroscopy (EDX) maps (Fig. 2c and f) of the fresh Ni_84_Cu_16_ and Ni_74_Cu_26_ catalysts, heated to the reaction temperature of 500 °C in Ar atmosphere. Additional EM images and EDX maps are shown in Fig. S7 and S8 (ESI†). Overall, the two metals were well mixed in the particles, yet some regions seemed to be more Cu or Ni-rich. Fig. S10 (ESI†) shows an evaluation of the copper content in particles with different sizes, to verify whether the Cu content was dependent on the particle size. Although larger particles tended to contain more copper, the variations in overall Cu content across the different as-prepared samples were large enough to outweigh the variations due to particle size. Furthermore, as the particles coalesce during reduction and catalysis, trends in the active catalyst nanoparticles are expected to be less pronounced.

### 3.2. Methane decomposition – macroscopic observations

**Fig. 3:**
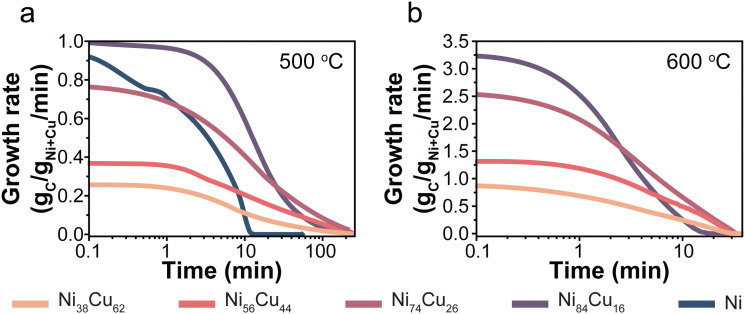
Evolution of the carbon growth rates over NiCu catalysts with varying Ni : Cu ratio at 500 (a) and 600 (b) °C. The Ni/C catalyst lost all activity instantly at 600 °C and is therefore excluded from the figure. 30% CH_4_/Ar, total pressure = 1 bar.

The catalysts were tested, in duplo, at 500 and 600 °C with 30% CH_4_/Ar in a thermogravimetric analyzer (TGA). For each catalyst, Fig. S2 (ESI†) shows the carbon yield as a function of time, including the individual duplo or triplo measurements. Fig. 3 presents the evolution of the carbon growth rate at 500 (a) and 600 °C (b). The monometallic Ni/C catalysts initially exhibited rapid growth, yet fully deactivated after 10 minutes at 500 °C. Unfortunately, the Ni/C at 600 °C hardly showed any carbon growth (Fig. S11, ESI†). The Cu-containing catalysts sustained the growth of carbon for significantly longer periods than the monometallic Ni-catalyst.

**Fig. 4:**
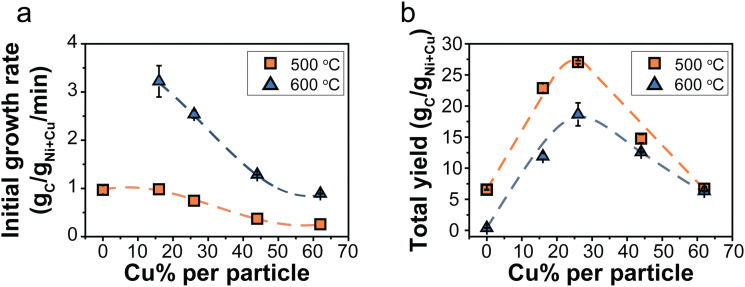
(a) Initial growth rate, *r*_0_, as a function of the Cu content (%) in the catalytst particles. (b) Final carbon yield obtained after 240 (500 °C) or 60 minutes (600 °C). Orange squares = 500 °C and blue triangles = 600 °C. Normalization per gram Ni reveals the same trend and is shown in Fig. S12 (ESI†).

It is interesting to evaluate both the trends in initial growth rates and final carbon yield. Table S3 (ESI†) lists *r*_0_ and the total carbon yield for all catalysts. The initial growth rates (*r*_0_) are shown in Fig. 4a. At 500 °C, the Ni/C and Ni_84_Cu_16_/C catalysts showed similar initial growth rates. Increasing the Cu content further in the catalyst particles, induced a decrease in initial growth rate. This effect is even more pronounced at 600 °C. For example, *r*_0_ is three times higher for catalysts containing 15–25% Cu with respect to 60% Cu. The initial growth rate of the Ni/C at 600 °C could not be properly determined as that catalyst deactivated very quickly.

Fig. 4b shows the final carbon yield at 500 and 600 °C after deactivation (240 or 60 minutes, respectively). The Ni/C catalyst yielded the lowest carbon due to quick deactivation. Introducing 15–25% Cu enhanced the carbon yield, but further increasing the Cu content beyond 30% led to a decrease. Catalysts containing 15–25% Cu showed similar initial growth rates at 500 °C as the Ni-catalyst but without rapid deactivation, resulting in longer sustained growth and, finally, more product. However, a higher Cu content slowed the reaction down, reducing the carbon yield. A maximum yield was achieved with 30% Cu. The influence of temperature was less pronounced for higher Cu contents.

Several explanations for the influence of Cu are reported in literature, such as improved methane adsorption,^15,17,23,26^ promoting carbon diffusion by an increased lattice constant,^23^ or stabilization of larger particles^27^ which provides more sustainable growth.^27,38^ Also, a dilution effect is mentioned: by the presence of inactive Cu at the catalyst surface, fewer active surface sites are available. Consequently, the reaction rate becomes slower.^26,29^ Yet, we postulate that yet another factor might play an important role: the solubility of carbon in Ni–Cu nanoparticles.^1^ Besides the ability to dissociate methane, one other reason that transition metals are used for this reaction is that these have a high carbon solubility.^1,22,23,48^

Nicholson determined the solubility of carbon in NiCu alloys with different Ni/Cu ratios at 1000 °C.^36^ It was shown that the solubility does not change in the range of 0–25% Cu, yet, exceeding 30 wt% Cu the carbon solubility steadily decreases to zero. This was assigned this to a correlation between the density of states at the Fermi level and the carbon solubility.

Stocks, Willams, and Faulkner calculated the density of states (DoS) of Ni–Cu alloys over the whole stoichiometric regime.^49^ For pure copper, the Fermi level (*E*_F_) is located at the top of the d-band, while for pure Ni the Fermi level is positioned within the d-band. Upon adding small amounts of Ni to Cu, there is a noticeable shift in both the shape and position of the d-band. By increasing the amount of Ni, a new band starts to form in the middle of the Cu d-band and *E*_F_. When the Ni content exceeds 40% the structure starts to resemble that of pure Ni. According to the work of Nicholson, the change of the DoS at the Fermi level explains the trend in the solubility of carbon as a function of Cu content in Ni–Cu alloys.^9^ The largest changes in DoS were observed between 40–60% Cu in the alloys.

**Fig. 5:**
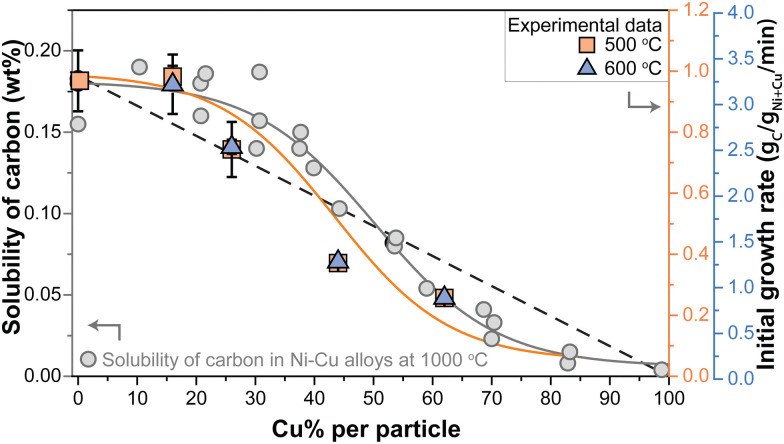
(left axis) Solubility of carbon (wt%) in Ni–Cu alloys with varying Cu content at 1000 °C as determined by Nicholson.^36^ Gray solid line indicates the trend. (right axis) Initial growth rates derived from the methane decomposition reactions at 500 °C (orange squares) and 600 °C (blue triagnles). Orange solid line indicates the trend. Dashed black line indicates an alternative model: the dilution effect.

In Fig. 5, the grey data points show the carbon solubility determined by Nicholson (left *y*-axis). To illustrate the effect of the solubility, the initial growth rates determined in our study are shown in orange squares (500 °C) and blue triangles (600 °C). The dashed line shows the expected trend of decreasing initial growth rate if solely caused by a dilution effect. As at 500 °C, we are operating in the regime where methane dissociation is limiting,^10^ a linear decrease should be expected. Clearly, the trend in *r*_0_ more closely resembles the trend in the carbon solubility with Cu content (orange solid line) determined by Nicholson. It should be noted that the solubility data were obtained at a somewhat higher temperature, although this is not expected to change the general trends. Interestingly, the same trend holds for the initial growth rates in the high-temperature regime (blue triangles).

The balance between carbon supply, transport, and nanostructure growth is clearly key for sustained carbon growth.^11,38^ The results described above suggest that diffusion of carbon through the metal nanoparticle is rate limiting, with carbon transport being the product of the concentration of mobile species and their mobility. The concentration gradient, the driving force for the mobility, build up between the surface of the nanoparticle where the carbon is dissolved due to methane decomposition, and the location where the carbon concentration is lower due to consumption at the metal/carbon filament interface.^50–52^ As the trend of the initial growth rate lies a bit below the trend of the solubility as a function of Cu content, it might be that this driving force slightly decreases due to a lower methane dissociation rate due to the dilution of Ni with Cu at the surface. However, overall the trends of initial growth rate and carbon solubility match very well, strongly suggesting that the decrease in carbon solubility, and hence the supply of carbon to the location of carbon nanostructure growth is the dominant factor.

The optimum in carbon yield can be explained as the addition of Cu initially keeps the active surface clear of too high concentrations of amorphous carbon and prevents catalyst encapsulation. However, excessive Cu addition significantly lowers the carbon solubility and hence growth rate, and consequently leads to a lower overall yield at high Cu concentrations.

Raising the temperature from 500 to 600 °C, not only raises the methane dissociation rate but also increases the carbon solubility in Ni particles.^53^ This leads to a higher saturation concentration and a larger number of carbon atoms in the system. Yet, the carbon precipitation rate, which is the limiting step in this high-temperature range,^10^ remains unchanged. Consequently, the driving force for diffusion diminishes, leading to carbon accumulating at the catalyst surface. Hence the fast deactivation at 600 °C (Fig. 3a).

### 3.3. A microscopic picture

**Fig. 6:**
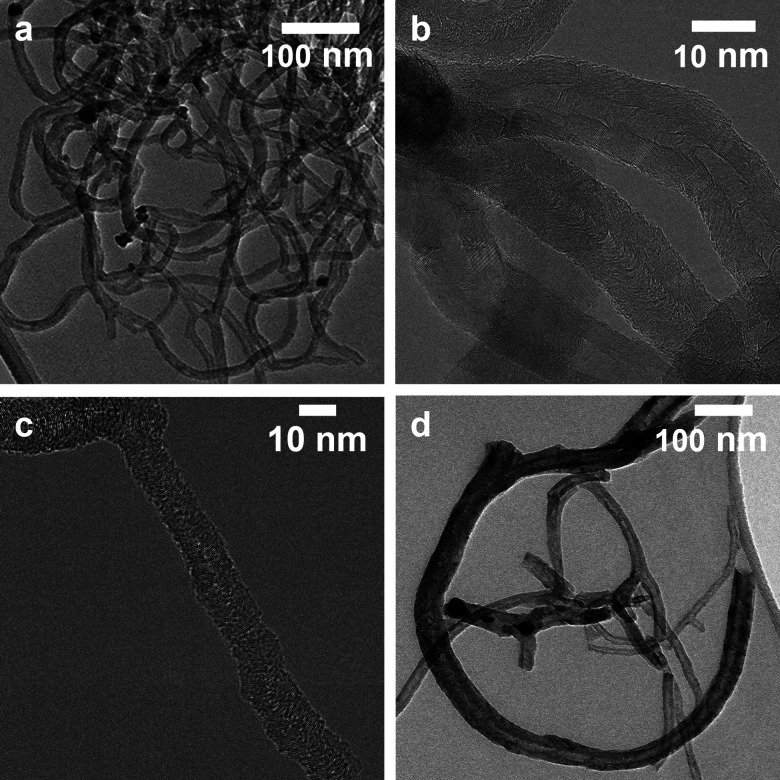
TEM images of the reaction products. (a) Carbon nanofibers grown over the Ni_74_Cu_26_ catalyst at 500 °C. (b) Hollow CNFs grown over the Ni_74_Cu_26_ catalyst at 600 °C. (c) Example of a full fiber grown over the Ni_38_Cu_62_ catalyst at 500 °C. (d) Fibers grown over the Ni_38_Cu_62_ catalyst at 500 °C. Example of fragmentation.

Transmission electron microscopy (TEM) images of the carbon nanofibers grown over the Ni_74_Cu_26_ and Ni_38_Cu_62_ catalysts are shown in Fig. 6. The average fiber diameters were evaluated based on the TEM images, they are listed in Table S3 (ESI†). The size distributions are shown in Fig. S13 (ESI†). Commonly, the fiber diameter is closely related to the size of the fiber they grew from. For all catalysts the average fiber diameter is larger than the catalyst particle size before catalysis. This indicates that during the reaction, catalyst metal nanoparticles grow, resulting in larger metal particles and thicker carbon fibers being grown out of them.

Two types of fibers were observed: hollow fibers (Fig. 6b) and full fibers (Fig. 6c). The fraction of hollow fibers is generally higher at 600 °C than at 500 °C for the NiCu catalysts, which is ascribed to a change in the rate-determining step for nanofiber growth.^10^ Methane dissociation is the limiting factor at low temperatures, while the carbon structure formation is limiting at higher temperatures – leading to more formation of hollow fibers. With increasing amounts of Cu in the samples, fragmentation of the metal particles is observed – especially at 600 °C (Fig. 6d). This is a known deactivation mechanism for Ni–Cu catalysts.^47,54^ Fragmentation mainly happens at higher temperatures when the catalyst particles become easy to deform. The fragmentation process is strongly influenced by the Ni/Cu ratio, as an increasing Cu content decreases the melting point of the nanoparticles.^47,54^

In the second part of this study, we zoom in on the individual particle level using *in situ* TEM. This technique can provide additional (essential) insights into the carbon nanostructure formation. Where the TGA experiments provide meaningful statistical data, *in situ* TEM provides information about individual particles, while being relevant for reactor-scale studies.^38^

We performed methane decomposition TEM experiments at atmospheric pressure at 600 °C. The gas composition was 30% CH_4_, 10% H_2_, and 60% Ar. Hydrogen gas was required for reliable imaging conditions.^38^ A schematic of the gas cell and the temperature profile is described in our previous work.^38^ After the gas cell was introduced in the microscope, the sample was first dried under Argon at 100 °C. Next, an additional *in situ* reduction was performed on the pre-reduced catalyst for 45 minutes in 5% H_2_ in Ar (300 °C for NiCu samples, and 330 °C for Ni samples). Next, the temperature was raised to 600 °C. The reaction gas was introduced as soon as the reaction temperature was reached. At this moment CNFs started growing as can be seen in Movie S1 (ESI†). Snapshots of different moments in time during the *in situ* TEM experiments are shown in Fig. S14 (ESI†).

**Fig. 7:**
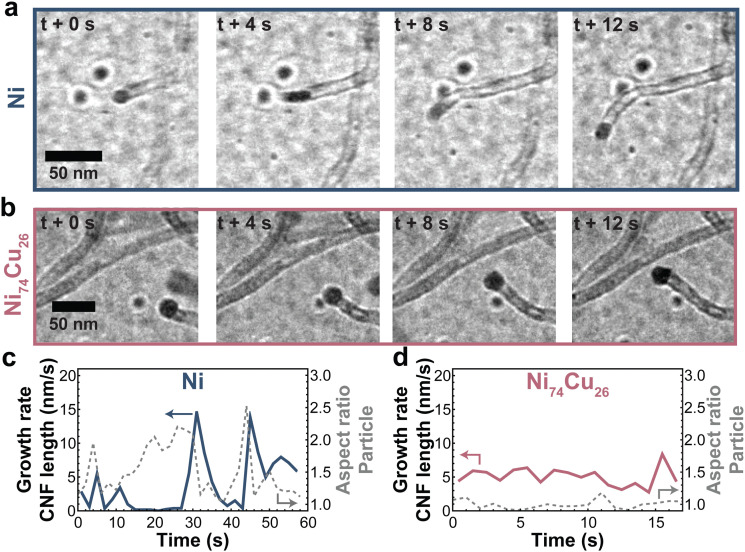
Typical CNF growth from Ni and NiCu particles. Snapshots of CNFs grown by a (a) Ni and (b) NiCu (26 wt% Cu) particle. (c and d) The CNF length growth rate (solid line) and particle aspect ratio (dashed line) for the Ni and NiCu particle. Additional measurements are showcased in Fig. S15–S17 (ESI†).

Zooming in on the carbon growth from individual particles gives more insight into the origin of the difference between Ni nanoparticles and those richer in Cu. Fig. 7a and b shows snapshots taken from CNF growth of single particles of pure Ni (a) and Ni_74_Cu_26_ (b). Examples of additional individual particles can be found in Fig. S15 (ESI†). There are two main differences in the CNF growth induced by the presence of Cu. First, the Ni particle shape fluctuates significantly in time, while the NiCu particles did not show this behavior (Fig. 7c and d and Fig. S16 and S17, ESI†). Interestingly, this behavior is opposite to what would be expected based on the lower melting temperature of Cu. Second, the carbon growth rate of the NiCu sample is relatively constant in time, while the carbon growth from the Ni particle stutters: sometimes growth stops, only to continue later. This is best appreciated in Movies S2 (Ni) and S3 (Ni_74_Cu_26_) (ESI†). In other words, Ni particles show a pulsed-like growth. This might arise from the formation of a thin amorphous carbon layer around the Ni particle. The Ni particle is (temporarily) deactivated, and no CNF is grown for some time. However, when encapsulation is not complete, the Ni particle can deform and “escape”. Now, fresh Ni surface is exposed for methane molecules to adsorb, leading to an increase of the fiber growth rate.^1,55^ The formation of the thin amorphous layer arises from a disbalance in the rates of carbon supply, transport, and CNF formation. By the addition of Cu (up to 25%), the formation of this amorphous layer is prevented leading to a smoother CNF growth. The smooth and stable CNF growth from NiCu catalysts results in the longer lifetime of these catalysts compared to Ni catalysts.

For larger particles and particles with a high percentage of Cu, multiple fibers grow from a single particle (Fig. S18, ESI†), which ensures smooth growth as the carbon concentration gradient in the particle remains relatively steep. Movie S4 (ESI†) shows a 60 nm NiCu particle growing multiple fibers. The smooth growth from NiCu particles and stuttering growth from Ni particles support our earlier discussion on the changing balance between carbon supply, transport, and nucleation.

## 4. Conclusion

The influence of copper concentration (0–62%) in carbon-supported bimetallic NiCu catalysts for methane decomposition was investigated. The gravimetric methane decomposition experiments provided operando data on the carbon yield, revealing that an alloy containing 30% Cu yielded an optimal carbon yield, balancing a reasonably high growth rate (related to the carbon solubility in the metal nanoparticle) with a long catalyst lifetime. The dilution of the Ni surface by Cu effectively keeps the active surface clear and prevents catalyst encapsulation. However, too much copper negatively impacts both the reaction rate and yield by greatly reducing carbon solubility. Ni particles were found to fluctuate in shape and facilitate stuttering CNF growth, probably due to reversible coverage of part of the nanoparticle surface by amorphous carbon deposits. On the other hand, particles containing 26 wt% Cu were larger, remained spherical and showed smooth CNF growth. Our findings underscore the importance of combining statistical and nanoscale techniques to understand the behavior of ensembles of nanoparticles and of balancing the Ni/Cu ratio to optimize catalyst performance and lifetime in methane decomposition.

## Author contributions

SES – conceptualization, formal analysis, methodology, validation, visualization, writing – original draft. SB – methodology, validation. DFLW – resources. JDM – resources. TAJW – visualization, resources, supervision, writing – original draft. PEdJ – funding acquisition, supervision, writing – review & editing.

## Conflicts of interest

There are no conflicts to declare.

## Supplementary Material

MA-005-D4MA00138A-s001

MA-005-D4MA00138A-s002

MA-005-D4MA00138A-s003

MA-005-D4MA00138A-s004

MA-005-D4MA00138A-s005
