# Supplementary material for: Balancing act: influence of Cu content in NiCu/C catalysts for methane decomposition
Source: Mater Adv. 2024 Mar 30;5(10):4251–61. doi: 10.1039/d4ma00138a (PMC11103560; doi:10.1039/d4ma00138a)
Supplement: MA-005-D4MA00138A-s005 [file MA-005-D4MA00138A-s005.pdf]

Electronic Supplementary Material (ESI) for Materials Advances.
This journal is © The Royal Society of Chemistry 2024

Supporting Information for

Balancing Act: Influence of Cu content in NiCu/C catalysts for methane decomposition

Suzan E. Schoemaker[a], Stefan Bismeijer[a], Dennie F.L. Wezendonk[a], Johannes D. Meeldijk[a,b], Tom A. J. Welling[a,#,*], Petra E. de Jongh[a,*]

[a] *Materials Chemistry and Catalysis, Debye Institute for Nanomaterial Science, Universiteit Utrecht,*

Universiteitsweg 99, 3584 CG Utrecht, The Netherlands

[b] *Electron Microscopy Center, Faculty of Science, Universiteit Utrecht, Universiteitsweg 99, 3584 CG Utrecht, The Netherlands*

* Corresponding authors: t.a.j.welling@uu.nl ; p.e.dejongh@uu.nl

[#] *Present address: Frontier Research Institute for Interdisciplinary Sciences, Department of Chemical Engineering, Tohoku University, Sendai, Miyagi 9808578, Japan.* t.a.j.welling@tohoku.ac.jp

Contents

S1. Catalytic set-up 2
S2. Reproducibility 3
S3. Characterization fresh catalysts 4
S4. Additional catalytic data 10
S5. Supporting Movies 17

S1. Catalytic set-up

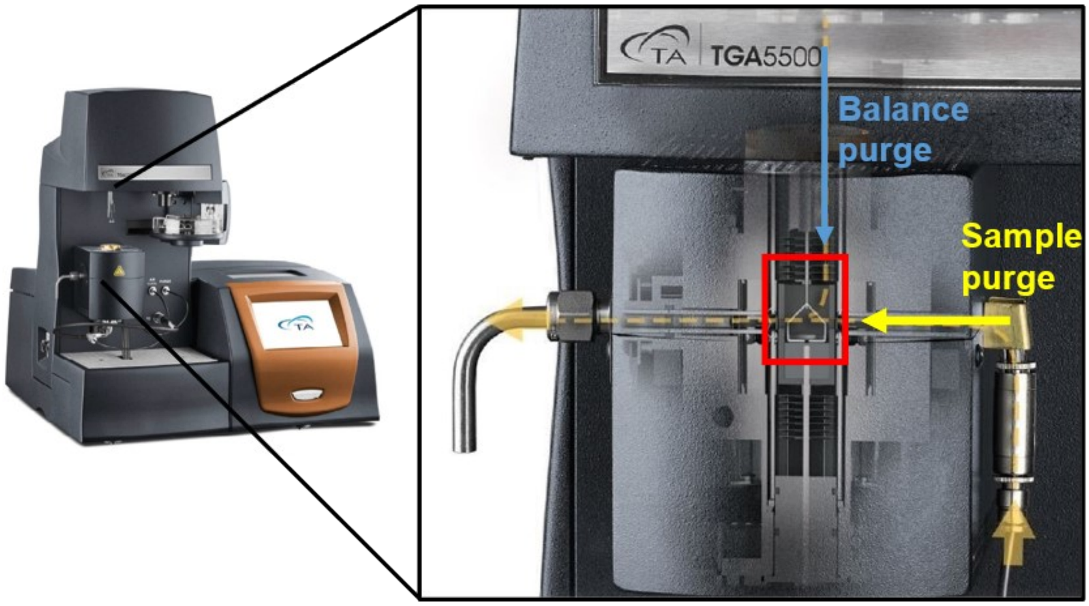

Figure S1. *Displayed on the left is an image of the TGA5500 from TA Instruments, equipped with an IR furnace, which was utilized as the catalytic setup in this study. On the right is a detailed view of the IR furnace. The location of the sample pan is clearly indicated in red. The gas flows within the system are marked by arrows. The system employs an argon balance purge (blue) to prevent any gases from entering the balance chamber. The sample purge (yellow), flowing horizontally over the sample, can be adjusted to a specific gas composition, ranging from pure argon to a mixture of hydrogen or methane in argon. During the experiments, the flow rate of the sample purge was maintained at twice that of the balance purge. It's important to note that the balance purge does not affect the gas composition surrounding the catalyst sample.*

Pictures were obtained from the TA Instruments website, https://www.tainstruments.com/tga-5500/. (visited at 01-12-2023)

S2. Reproducibility

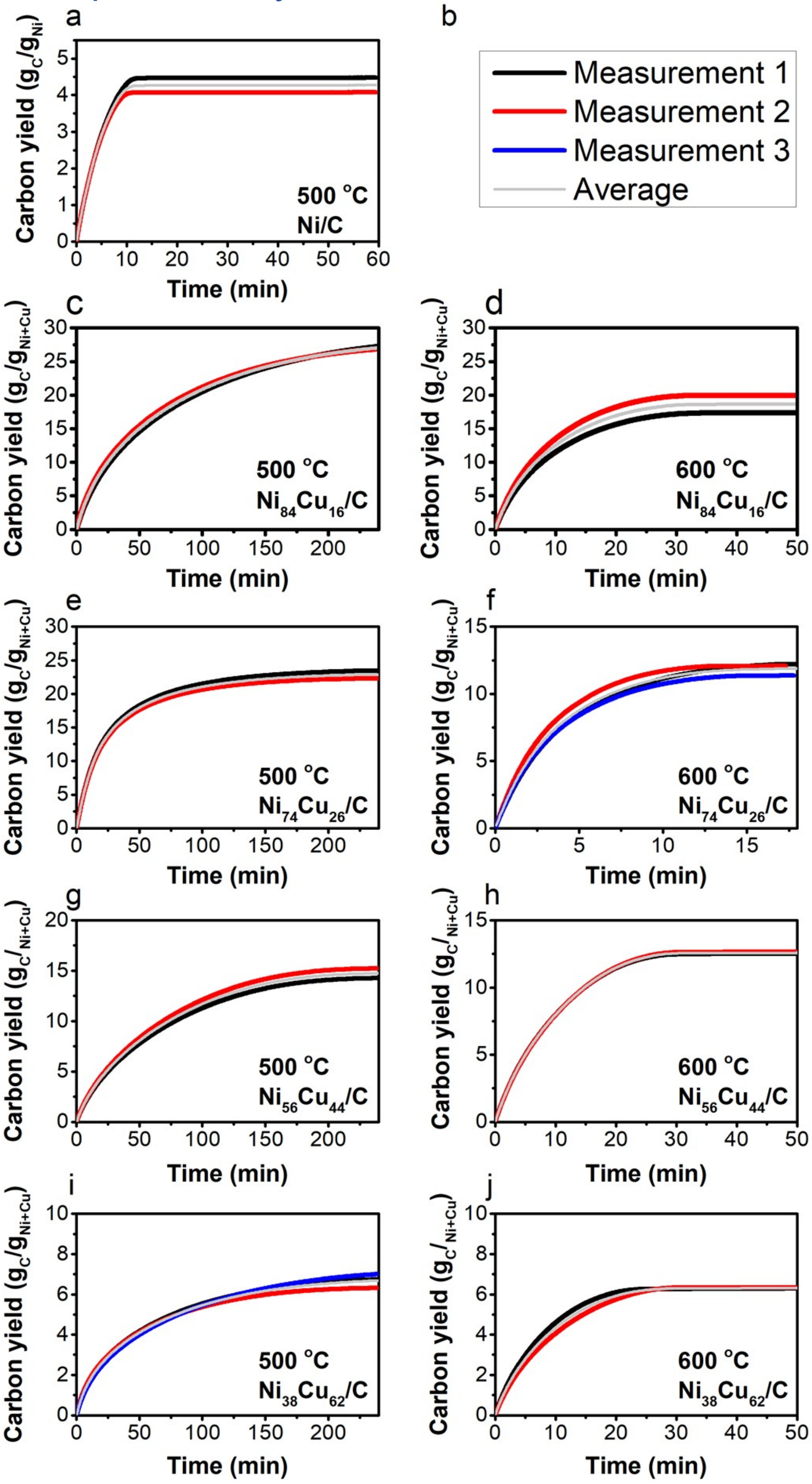

Figure S2 *All catalytic experiments conducted in the TGA were performed in duplicate, some were carried out in triplicate. The individual experiments, each normalized per gram of metal, are displayed. The normalization accounts for any variations in sample loading. The averaged results of these experiments are depicted in gray. Left: 500 °C, right 600 °C. a. Ni/C, c,d.*$Ni_{84}Cu_{16}$*, e,f.* $Ni_{74}Cu_{26}$*, g,h.* $Ni_{56}Cu_{44}$ *i,j.* $Ni_{38}Cu_{62}$.

S3. Characterization fresh catalysts

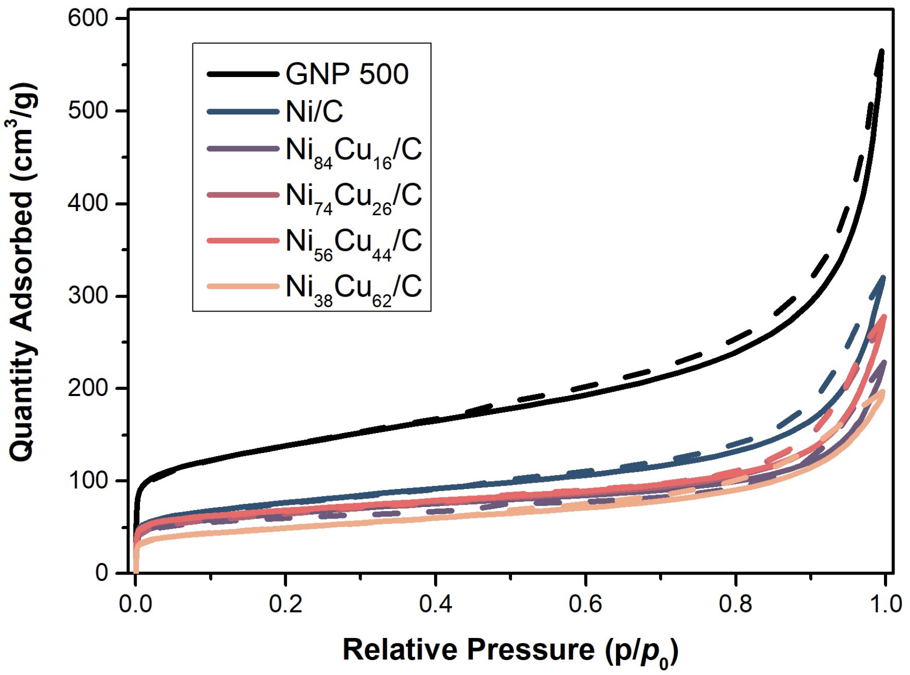

Figure S3 *N_2 physisorption of the selected catalysts used and the support (GNP 500) measured at 77K. Solid line = adsorption isotherm, dotted line = desorption line. The BET surface area was determined in the relative pressure range of p/p_0 = 0.02-0.1. The total pore volume was determined from the adsorbed quantity at p/p_0=0.995.*

Table S1 *BET surface area and total pore volume determined by N_2-physisorption*

Sample	BET surface area (m^2/g)	Total pore volume (cm^3/g)
**GNP 500**	494	0.87
**Ni/C**	273	0.48
**$Ni_{84}Cu_{16}/C$**	234	0.34
**$Ni_{74}Cu_{26}/C$**	243	0.41
**$Ni_{56}Cu_{44}/C$**	249	0.42
**$Ni_{38}Cu_{62}/C$**	174	0.30

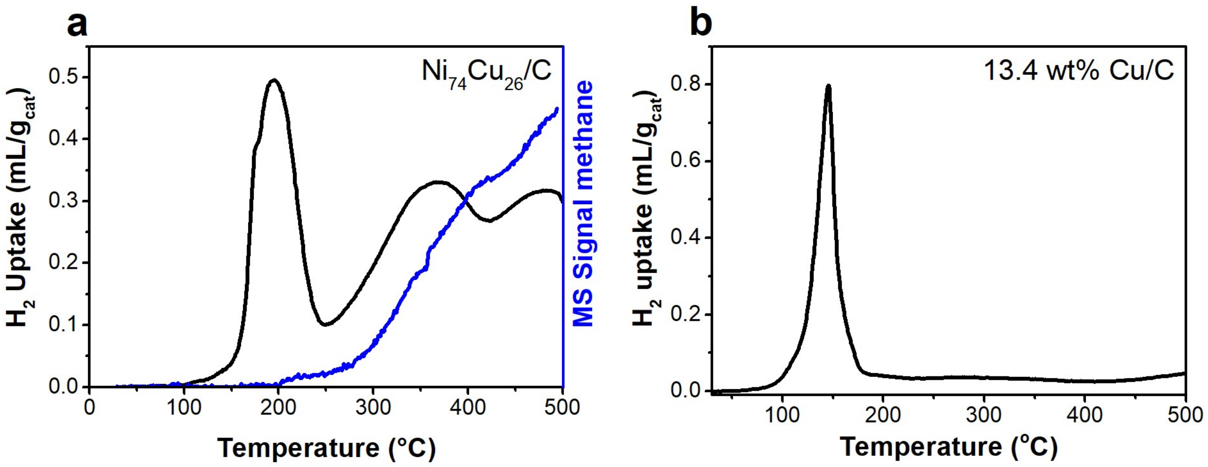

Figure S4 a. *Reduction profile (black) and corresponding methane MS signal (blue) of the* $Ni_{74}Cu_{26}$*/C catalyst. Support methanation becomes significant above 300 °C. b. Reduction profile of a reference Cu/C catalyst (13.4 wt%).[1] 5 °C* min^{-1} *in 5%* H_2*.*

[1] S.E. Schoemaker, T.A.J. Welling, D.F.L. Wezendonk, B.H. Reesink, A.P. van Bavel, P.E. de Jongh, Carbon nanofiber growth from methane over carbon-supported NiCu catalysts: Two temperature regimes, Catal Today. 418 (2023). https://doi.org/10.1016/j.cattod.2023.114110.

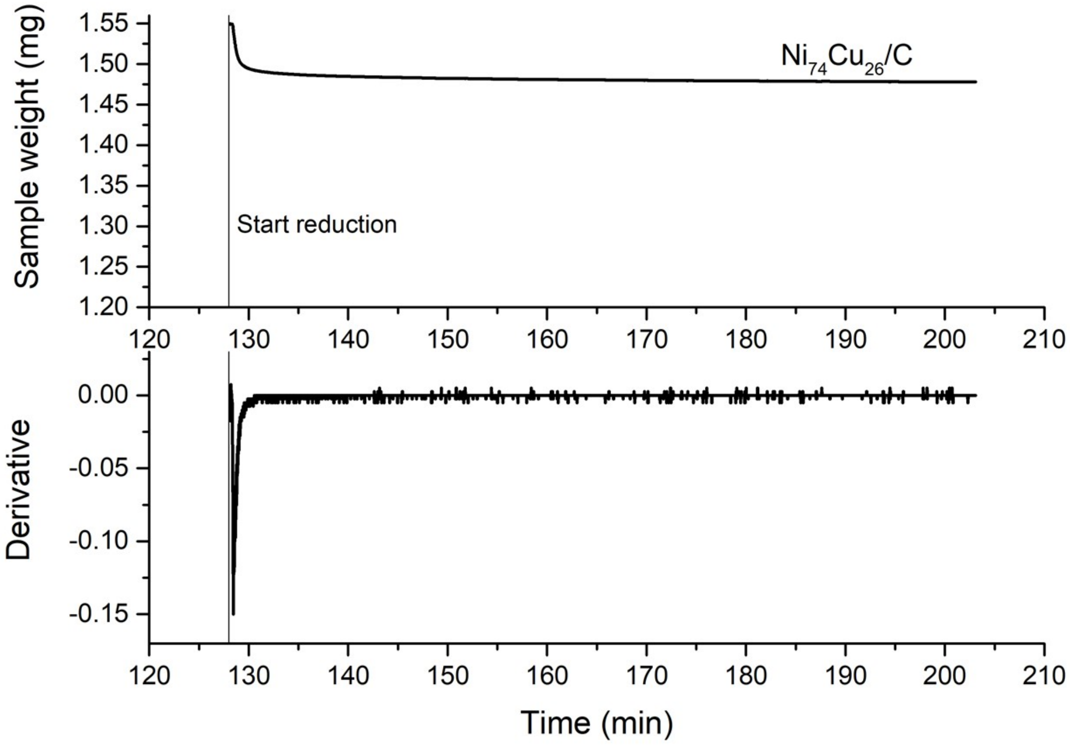

Figure S5 *Sample weight change during the in-situ reduction step (300 °C) of the* $Ni_{74}Cu_{26}$*/C catalyst prior to catalytic reaction inside the TGA (top) and the derivative over time (bottom).*

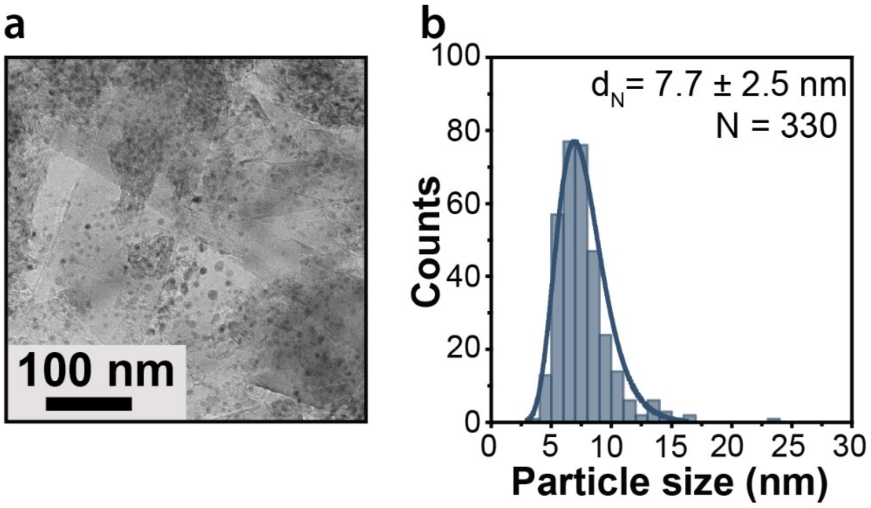

Figure S6 a. *TEM image of Ni/C catalyst fresh at 500 °C. b. Corresponding size distribution.*

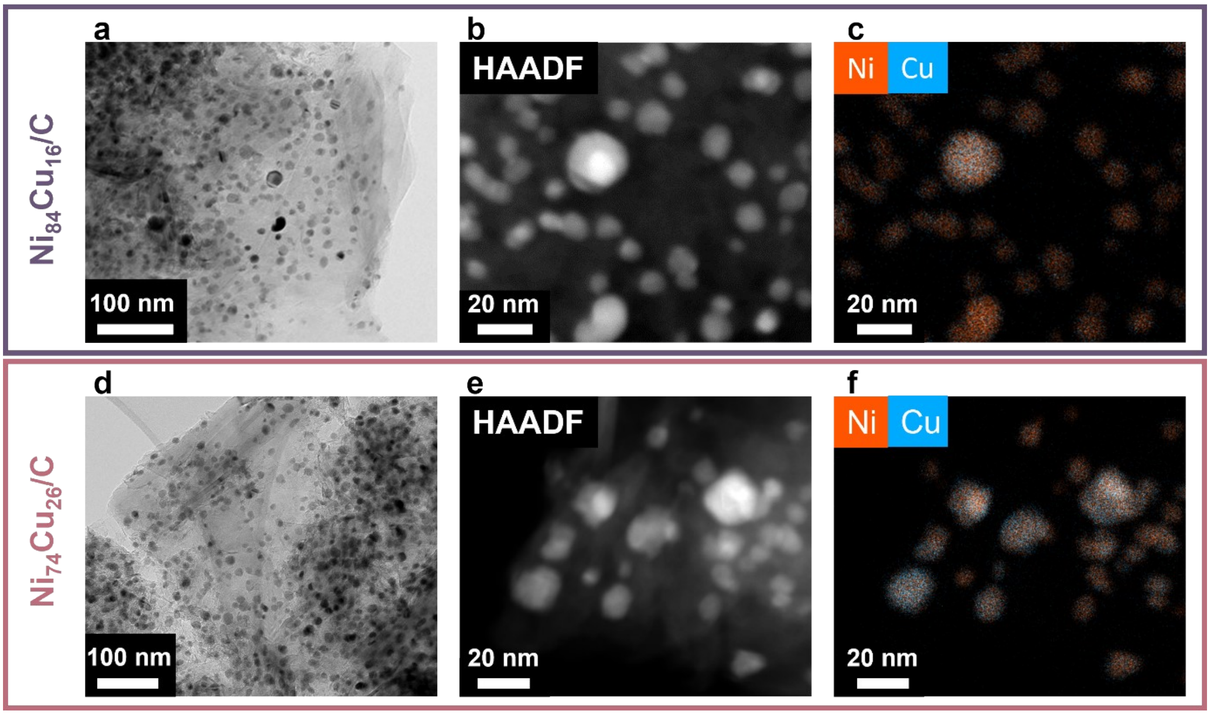

Figure S7 *Transmission electron microscopy images (left), HAADF STEM (middle) and corresponding EDX maps (right) of the fresh $Ni_{84}Cu_{16}$ (a-c) and $Ni_{74}Cu_{26}$ (d-f) catalysts before reaction at 600 °C.*

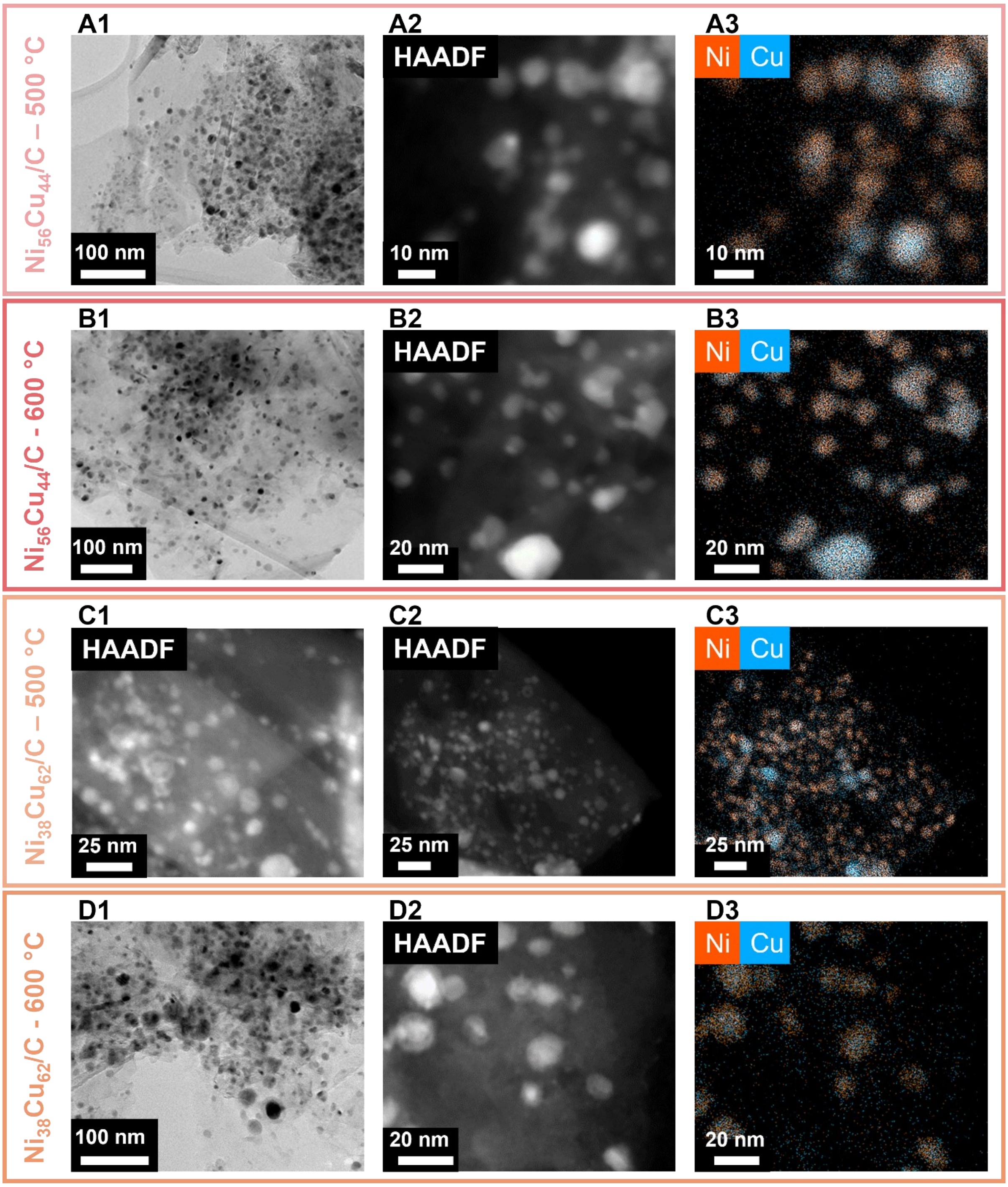

Figure S8 *Transmission electron microscopy images, HAADF STEM and corresponding EDX maps of the fresh* $Ni_{56}Cu_{44}$ *(frame A + B) and* $Ni_{38}Cu_{62}$ *(frame C + D) catalysts before reaction.*

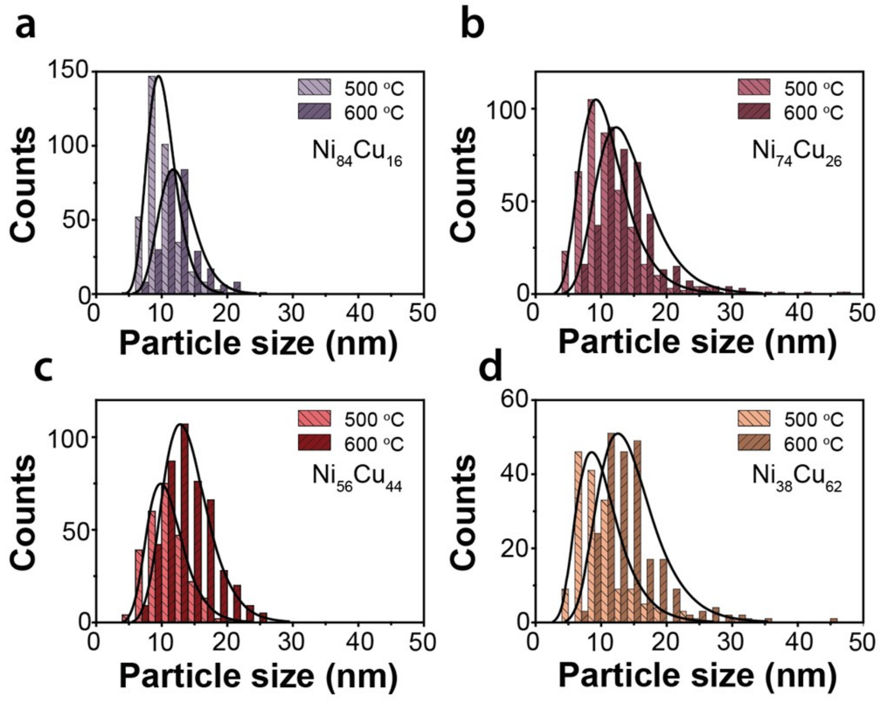

Figure S9 *Particle size distributions of the selected catalysts determined TEM.*

Table S2 *Average particle sizes determined by TEM.*

	**Sample**	**Particle size fresh catalyst (nm)**	
		500 °C	600 °C
**1**	**Ni/C**	7.7 ± 2.5 (n=330)	-
**2**	**$Ni_{84}Cu_{16}$/C**	10.2 ± 2.0 (n = 363)	12.6 ± 3.0 (n = 267)
**3**	**$Ni_{74}Cu_{26}$/C**	11.0 ± 4.0 (n = 413)	14.3 ± 5.0 (n = 390)
**4**	**$Ni_{56}Cu_{44}$/C**	10.9 ± 3.0 (n = 263)	14 ± 4.0 (n = 449)
**5**	**$Ni_{38}Cu_{62}$/C**	10.4 ± 4.5 (n = 162)	14.7 ± 6.0 (n = 232)

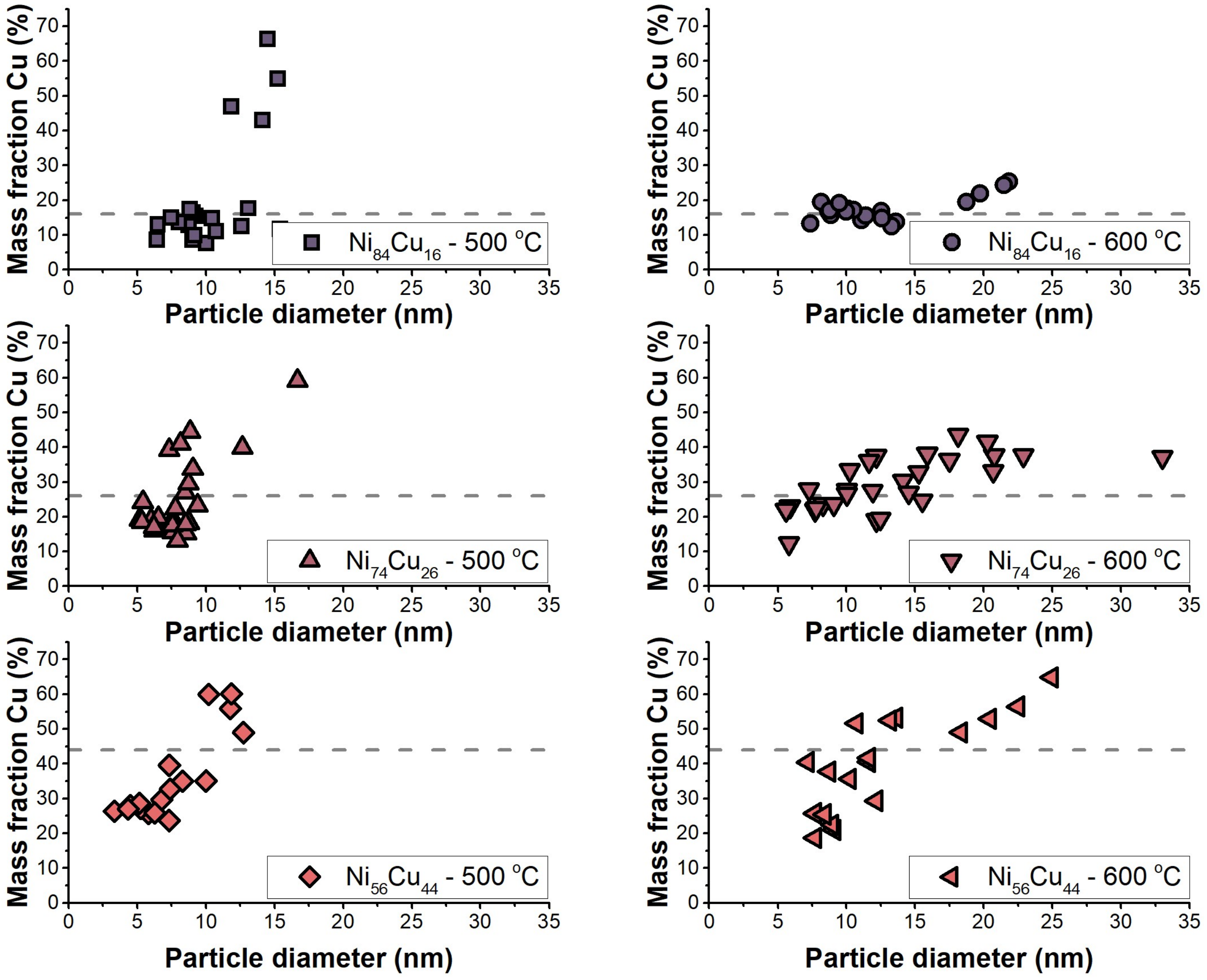

Figure S10 *Copper mass fraction in multiple particles after reduction determined by STEM-EDX as function of the particle size. Dashed line: average mass fractions as determined by ICP are 16% (top), 26% (middle) and 44%(bottom). Please note that a larger sample size is required to acquire statistically significant information regarding the Cu weight loading in the particles using STEM-EDX. Nevertheless, if a distinct correlation exists between particle size and Cu content, it should already be evident in the current data.*

S4. Additional catalytic data

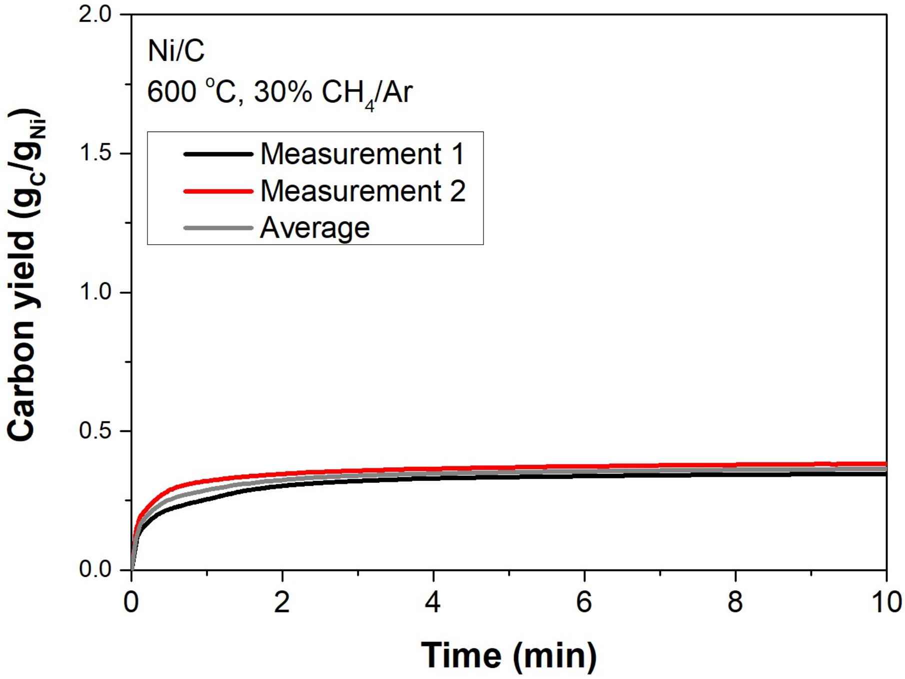

Figure S11 *Methane decomposition experiment in the TGA over Ni/C catalyst at 600 °C with 30% CH_4 in Ar. Individual measurements shown in black and red, average in gray.*

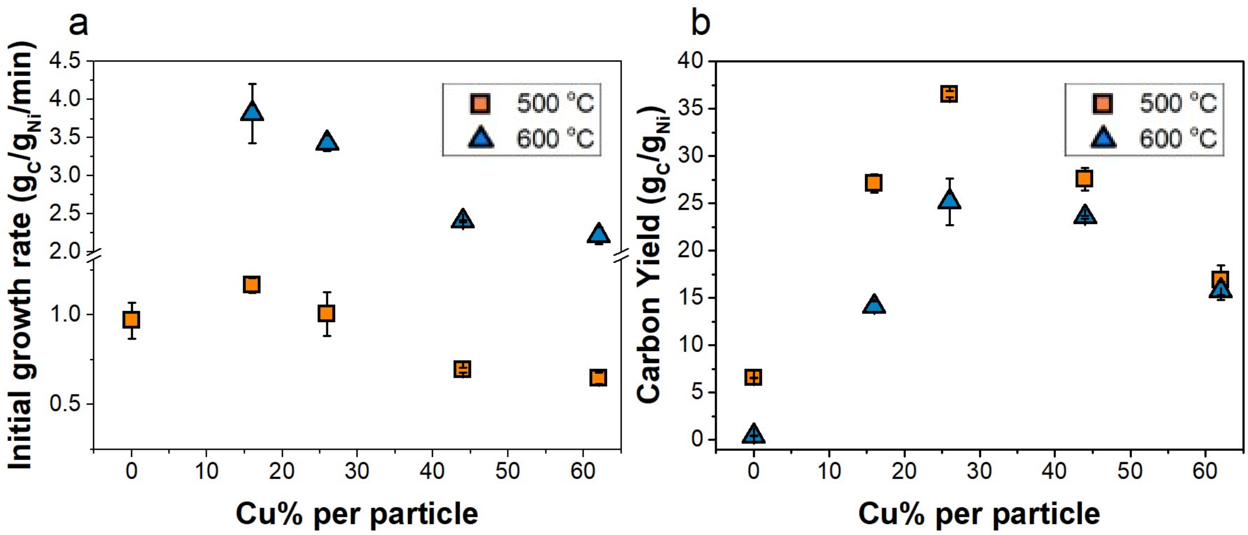

Figure S12 a*. Initital growth rate, r0, as a function of the amount of Cu in the catalytst particles. b. Final carbon yield obtained after reaction time for all tested catalysts.Orange squares = 500 °C and blue triangles = 600 °C. Both normalized per gam Ni showing the same trend as Figure 4 in the main text.*

Table S3 *Initial growth rates, total carbon yield and average fiber diameter observed during the methane decomposition experiments in the TGA.*

Sample	Initial growth rate (g_c/g_{Ni+cu}/min)		Total carbon yield (g_c/g_{Ni+cu})		Average fiber diameter (nm)	
	500 °C	600 °C	500 °C	600 °C	500 °C	600 °C
**Ni/C**	0.97± 0.1	-	6.55 ± 0.04	0	18.5 ± 9.6 (n=97)	-
**$Ni_{84}Cu_{16}$/C**	0.98 ± 0.04	3.22 ± 0.32	22.9 ± 0.8	11.9 ± 0.5	15.5 ± 3.7 (n = 59)	16.1 ± 4.3 (n = 81)
**$Ni_{74}Cu_{26}$/C**	0.74 ± 0.09	2.54 ± 0.08	27.0 ± 0.2	18.6 ± 1.8	22.8 ± 11.5 (n = 135)	22.1 ± 7.1 (n = 122)
**$Ni_{56}Cu_{44}$/C**	0.37 ± 0.01	1.285 ± 0.003	14.8 ± 0.7	12.6 ± 0.1	17.7 ± 6.1 (n = 53)	23.5 ± 7.5 (n = 49)
**$Ni_{38}Cu_{62}$/C**	0.25 ± 0.01	0.890 ± 0.004	6.7 ± 0.3	6.3 ± 0.04	19.6 ± 7.0 (n = 90)	20.5 ± 8.5 (n = 93)

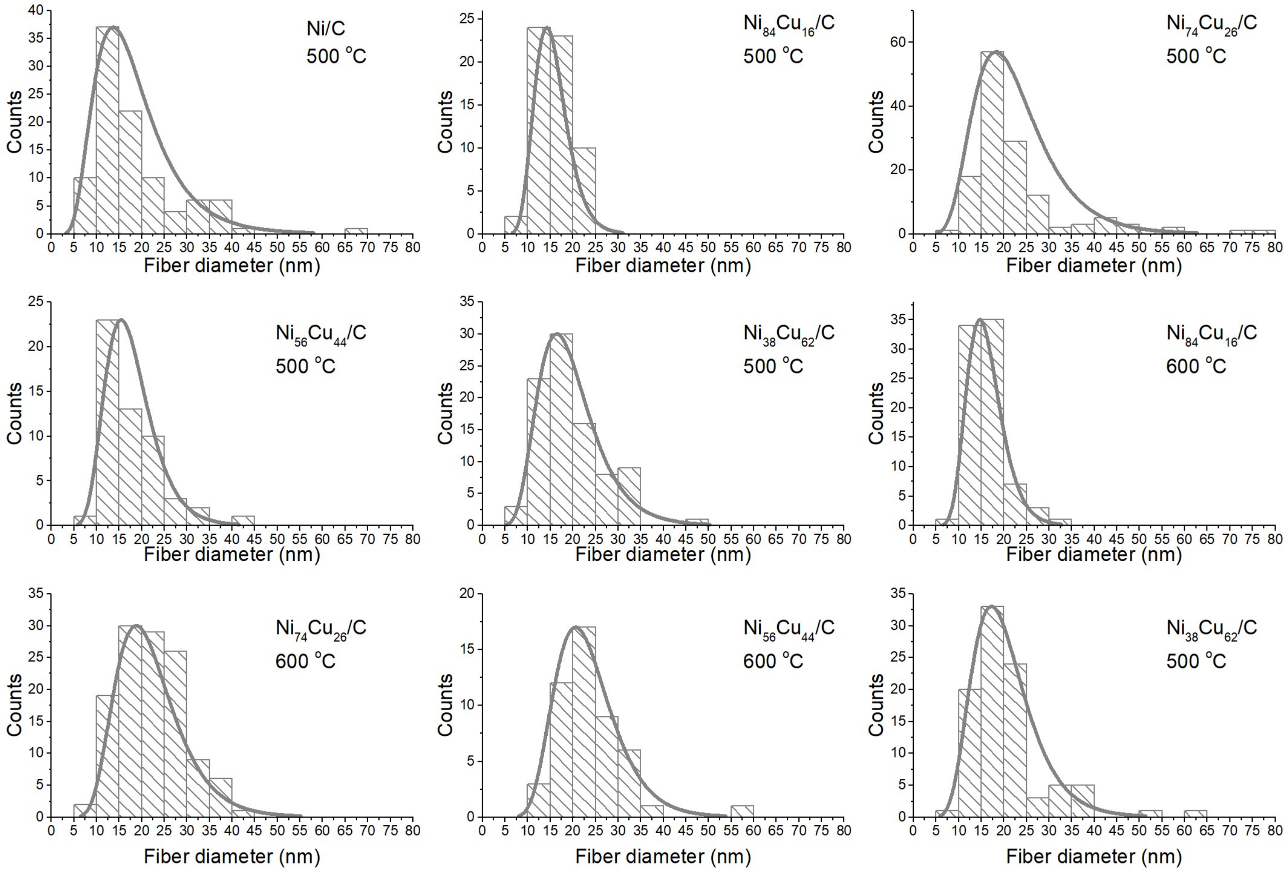

Figure S13 *Distribution of fiber diameters grew from the different catalysts at 500 °C or 600 °C determined with TEM. Average diameters are listed in Table S4.X.*

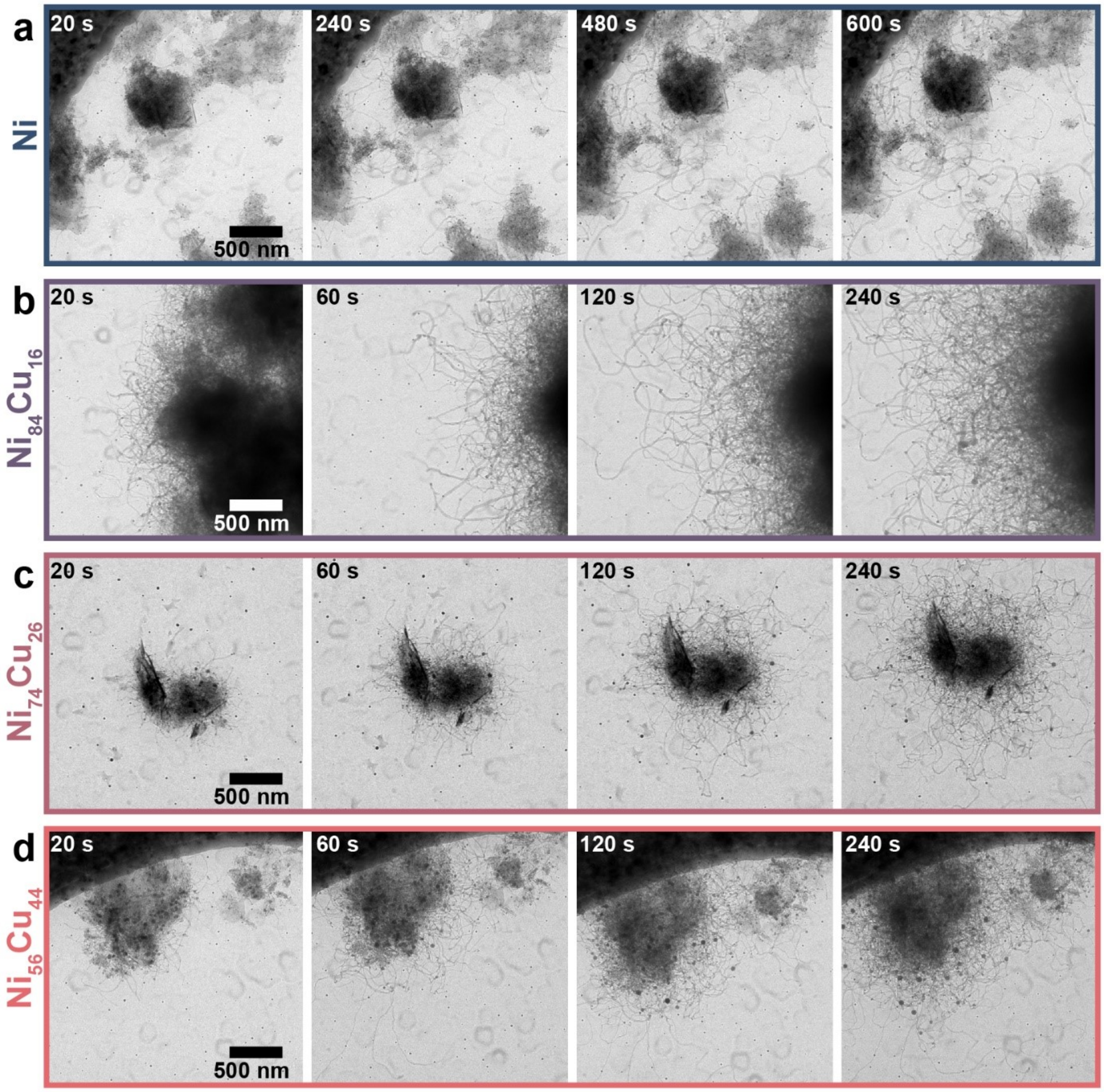

Figure S14 *Snapshots of different moments in time during additional in-situ gas-cell TEM experiments at atmospheric pressure at 600 °C. The gas composition was 75%* CH_4 *and 25%* H_2.

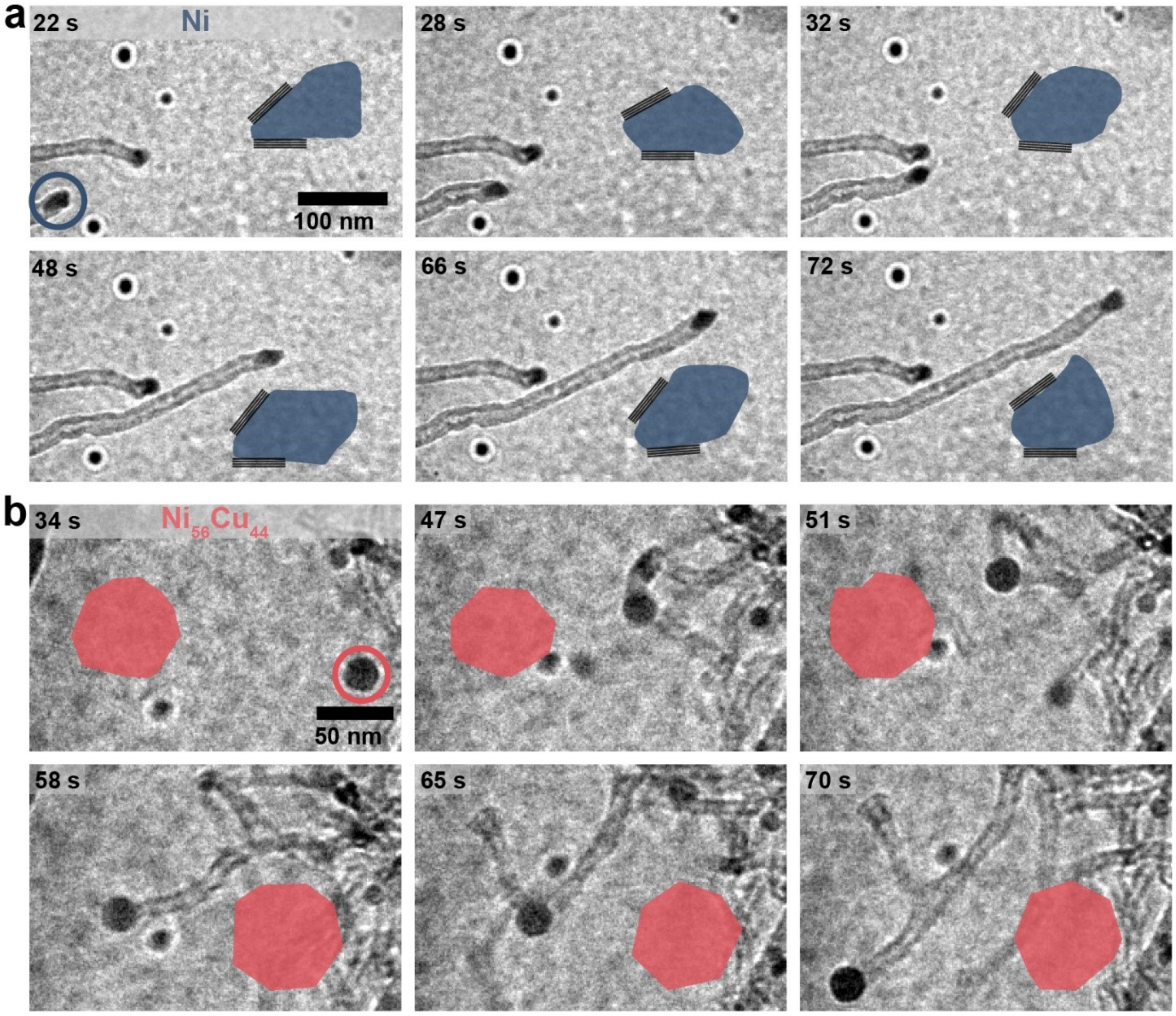

Figure S15 *Snapshots during the in-situ TEM experiments for (a) pure Ni and (b)* $Ni_{56}Cu_{44}$ *catalysts. The particle shape of one particle is shown at each time interval. Original particle is indicated in the first frames. The Ni particle changes shape while the bimetallic particle is not.*

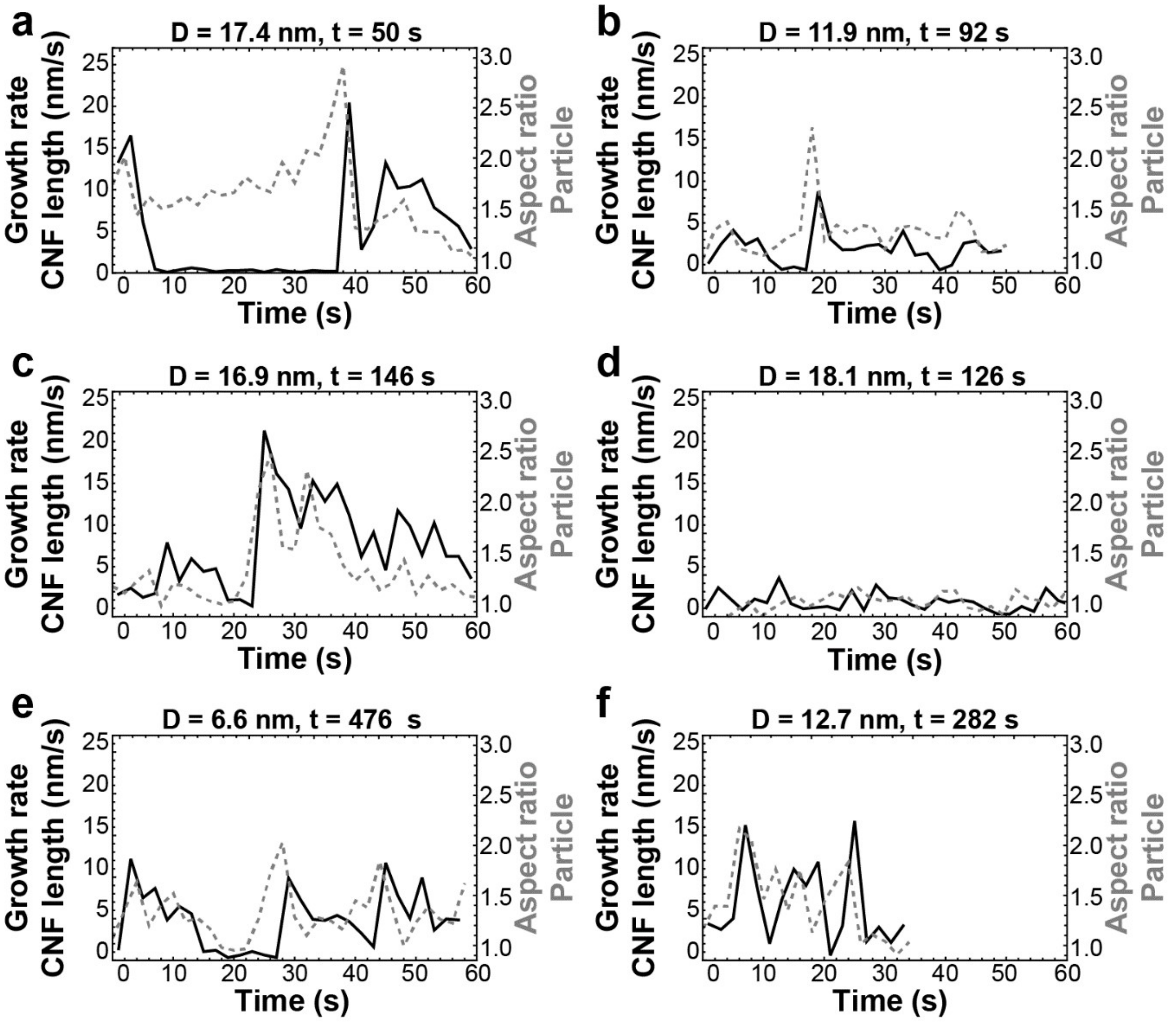

Figure S16 *The CNF length growth rate (solid line) and particle aspect ratio (dashed line) for multiple Ni particles. The metal particle sizes and time after the reaction started are indicated on top of each frame.*

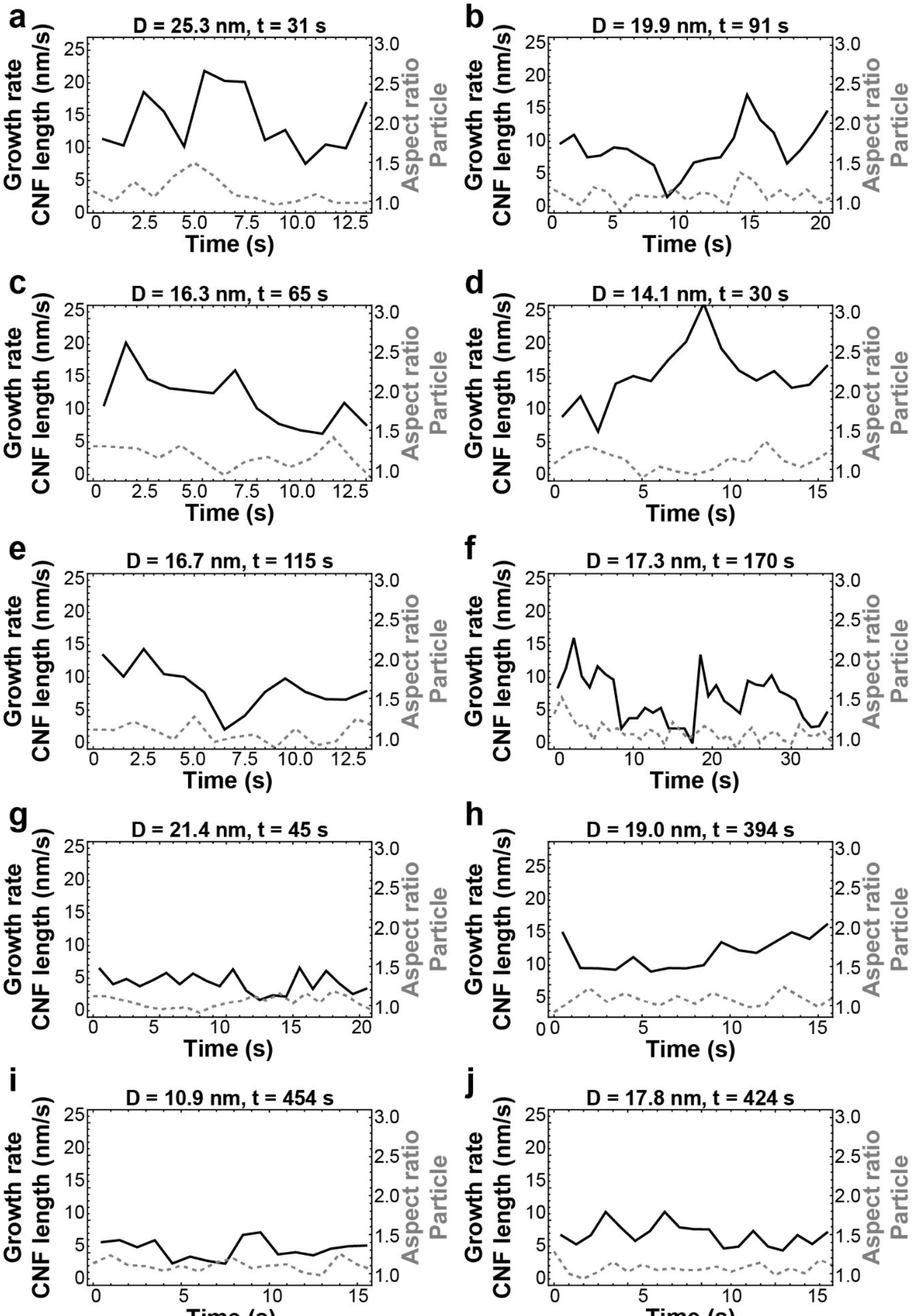

Figure S17 *The CNF length growth rate (solid line) and particle aspect ratio (dashed line) for multiple NiCu particles of the* $Ni_{74}Cu_{26}$*/C catalyst. The metal particle sizes and time after the reaction started are indicated on top of each frame.*

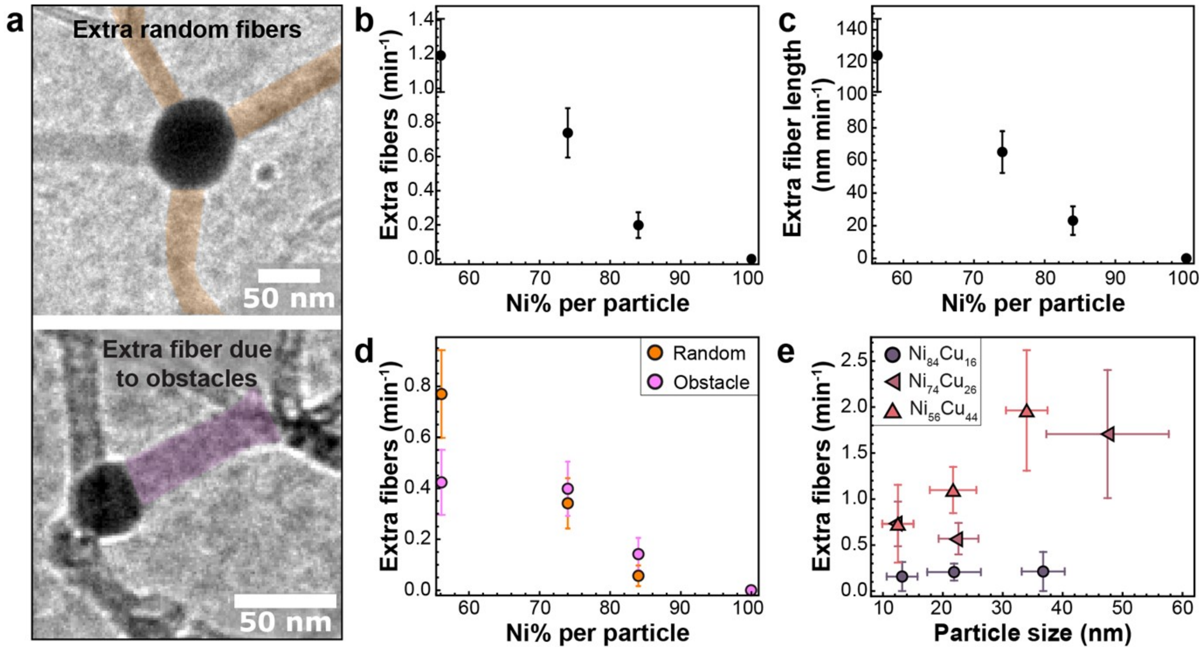

Figure S18 a. *Additional fiber formation occurring spontaneously (top) or as a result of encountering obstacles (bottom). b-d. Relation between the Ni wt% per particle and the number of extra fibers formed per minute (b); the growth rate of the extra fibers (nm min*$^{-1}$*) (c); The number of fibers formed randomly (orange) or due to encountering an obstacle (pink) (d); e. The number of extra fibers formed as a function of particle size for CNF growth for the catalysts containing 16%, 26% or 44% Cu.*

S5. Supporting Movies

1. **Movie S1** Overview of carbon fibers growing from the $Ni_{74}Cu_{26}/C$ catalyst. The gas-cell TEM experiment was performed at atmospheric pressure at 600 °C. The gas composition was 30% CH_4, 10% H_2, and 60% Ar. Electron dose rate: 10 $e^{-}Å^{-2}s^{-1}$.
2. **Movie S2** Typical CNF growth on single Ni-particle used for **Figure 7a**. The gas-cell TEM experiment was performed at atmospheric pressure at 600 °C. The gas composition was 30% CH_4, 10% H_2, and 60% Ar. Electron dose rate: 10 $e^{-}Å^{-2}s^{-1}$.
3. **Movie S3** Typical CNF growth on single NiCu (26% Cu) particle used for **Figure 7b**. The gas-cell TEM experiment was performed at atmospheric pressure at 600 °C. The gas composition was 30% CH_4, 10% H_2, and 60% Ar. Electron dose rate: 10 $e^{-}Å^{-2}s^{-1}$.
4. **Movie S4** *Tentacle* CNF growth over a 60 nm NiCu particle. The gas-cell TEM experiment was performed at atmospheric pressure at 600 °C. The gas composition was 30% CH_4, 10% H_2, and 60% Ar. Electron dose rate: 10 $e^{-}Å^{-2}s^{-1}$.
